# Supplementary material for: A protein quality control pathway at the mitochondrial outer membrane
Source: eLife. 2020 Mar 2;9:e51065. doi: 10.7554/eLife.51065 (PMC7136024; doi:10.7554/eLife.51065)
Supplement: Supplementary file 1. [file elife-51065-supp1.docx]

| **Key Resources Table** | | | | |
| --- | --- | --- | --- | --- |
| **Reagent type (species) or resource** | **Designation** | **Source or reference** | **Identifiers** | **Additional i­nformation** |
| strain, strain background (*Saccharomyces cerevisiae*) | WCG4-11/21a | (Heinemeyer et al., 1991) | *ura3-5Δ leu2-3,112 his3-11,15 pre1-1 pre2-2* |  |
| strain, strain background (*Saccharomyces cerevisiae*) | WCG4a | (Heinemeyer et al., 1991) | *ura3-5Δ leu2-3,112 his3-11,15* |  |
| strain, strain background (*Saccharomyces cerevisiae*) | *UFD1* | *(Ye et al., 2001)* | *his4-519 ura3-52 ade1-100 leu2-3,112 UFD1* |  |
| strain, strain background (*Saccharomyces cerevisiae*) | *ufd1-1* | *(Ye et al., 2001)* | *his4-519 ura3-52 ade1-100 leu2-3,112 ufd1-1* |  |
| strain, strain background (*Saccharomyces cerevisiae*) | *NPL4* | *(Ye et al., 2001)* | *ura3-52 leu2∆1 trp1∆63 NPL4* |  |
| strain, strain background (*Saccharomyces cerevisiae*) | *npl4-1* | *(Ye et al., 2001)* | *ura3-52 leu2∆1 trp1∆63 npl4-1* |  |
| strain, strain background (*Saccharomyces cerevisiae*) | *CDC48* | *(Ye et al., 2001)* | *ura3-52 leu2-3,112 ade2-1 trp-1 his3 CDC48* |  |
| strain, strain background (*Saccharomyces cerevisiae*) | *cdc48-3* | *(Ye et al., 2001)* | *ura3-52 leu2-3,112 ade2-1 trp-1 his3 cdc48-3* |  |
| strain, strain background (*Saccharomyces cerevisiae*) | *CIM* | *(Ghislain et al., 1993)* | *ura3-52 lys2-801 ade2-101 trp1Δ63 his3Δ200 leu2Δ1 CIM* |  |
| strain, strain background (*Saccharomyces cerevisiae*) | *cim3-1* | *(Ghislain et al., 1993)* | *ura3-52 lys2-801 ade2-101 trp1Δ63 his3Δ200 leu2Δ1 cim3-1* |  |
| strain, strain background (*Saccharomyces cerevisiae*) | *SSA1* | (Becker et al., 1996) | *his3-11,15 leu2-3, 112 ura3-52 trp1-∆1 lys2 SSA1 ssa2-1::LEU2 ssa3-1::TRP1 ssa4-2::LYS2* |  |
| strain, strain background (*Saccharomyces cerevisiae*) | *ssa1-45^ts^* | (Becker et al., 1996) | *his3-11,15 leu2-3, 112 ura3-52 trp1-∆1 lys2 ssa1-45^ts^ ssa2-1::LEU2 ssa3-1::TRP1 ssa4-2::LYS2* |  |
| strain, strain background (*Saccharomyces cerevisiae*) | *uba1-204* | (Ghaboosi and Deshaies, 2007) | *can1-100 leu2-3,-112 his3-11,-15 trp1-1 ura3-1 ade2-1 uba1::KanMX [pRS313 CEN HIS3 uba1-204]* |  |
| strain, strain background (*Saccharomyces cerevisiae*) | *HSP82 HSC82* WT | *(Nathan and Lindquist, 1995)* | *ade2-1, leu2-3, 112, his3-11, 15, trp1-1, ura3-1, can1-100* |  |
| strain, strain background (*Saccharomyces cerevisiae*) | *hsc82Δ hsp82-G313N* | *(Nathan and Lindquist, 1995)* | *ade2-1, leu2-3, 112, his3-11, 15, trp1-1, ura3-1, can1-100, hsc82::LEU2 hsp82::LEU2 [pTGPD CEN TRP1 HSP82]* |  |
| strain, strain background (*Saccharomyces cerevisiae*) | SM5186 | *(Metzger et al., 2008)* | *his3Δ leu2Δ ura3Δ* rad23::*kanMX dsk2::kanMX* |  |
| strain, strain background (*Saccharomyces cerevisiae*) | SM5360 | *(Metzger and Michaelis, 2009)* | *his3Δ leu2Δ ura3Δ doa10::NatMX::HIS3 hrd1::KanMX* |  |
| strain, strain background (*Saccharomyces cerevisiae*) | SM5364 | *(Metzger et al., 2008)* | *his3Δ leu2Δ ura3Δ ubc6::HIS3 ubc7::NatMX* |  |
| strain, strain background (*Saccharomyces cerevisiae*) | SM5770 | *(Heck et al., 2010)* | *his3Δ leu2Δ ura3Δ met15Δ ubr1::KanMX san1::NatMX* |  |
| strain, strain background (*Saccharomyces cerevisiae*) | *rsp5-1* | *(Davierwala et al., 2005)* | *his3Δ1 leu2Δ ura3Δ0 met15Δ0 rsp5-1::KanMX* |  |
| strain, strain background (*Saccharomyces cerevisiae*) | *rsp5-3* | *(Davierwala et al., 2005)* | *his3Δ1 leu2Δ ura3Δ0 met15Δ0 rsp5-1::KanMX* |  |
| strain, strain background (*Saccharomyces cerevisiae*) | 5X EMC WT | *(Lahiri et al., 2014)* | *his3Δ leu2Δ ura3Δ met15Δ* |  |
| strain, strain background (*Saccharomyces cerevisiae*) | 5x EMC (YSL9) | *(Lahiri et al., 2014)* | *his3Δ leu2Δ ura3Δ met15Δ emc1::HIS5 emc2::hygMX4 emc3::HIS5 emc5::kanMX4 emc6::kanMX4* |  |
| strain, strain background (*Saccharomyces cerevisiae*) | yMM36 | *(Metzger et al., 2017)* | *his3Δ leu2Δ ura3Δ met15Δ SAM35HA::KanMX* |  |
| strain, strain background (*Saccharomyces cerevisiae*) | yMM37 | This study | *his3Δ leu2Δ ura3Δ met15Δ sam35-2HA^ts^::KanMX* |  |
| strain, strain background (*Saccharomyces cerevisiae*) | yMM40 | *(Metzger et al., 2017)* | *his3Δ leu2Δ ura3Δ met15Δ SEN2HA::KanMX* |  |
| strain, strain background (*Saccharomyces cerevisiae*) | yMM41 | *(Metzger et al., 2017)* | *his3Δ leu2Δ ura3Δ met15Δ sen2-1HA^ts^::KanMX* |  |
| strain, strain background (*Saccharomyces cerevisiae*) | yMM149 | This study | *ura3-5Δ leu2-3,112 his3-11,15 ubr1::KanMX* |  |
| strain, strain background (*Saccharomyces cerevisiae*) | yJS155 | This study | *his3Δ leu2Δ ura3Δ met15Δ ubx2::KanMX* |  |
| strain, strain background (*Saccharomyces cerevisiae*) | yJS208 | This study | *ura3-5Δ leu2-3,112 his3-11,15 doa1::KanMX* |  |
| strain, strain background (*Saccharomyces cerevisiae*) | *Ubx2-GFP* | Thermo-Fisher Scientific | *his3Δ leu2Δ ura3Δ met15Δ UBX2-GFP(S65T)::HIS3MX6* |  |
| strain, strain background (*Saccharomyces cerevisiae*) | *Ubx2-TAP* | GE Dharmacon | *his3Δ leu2Δ ura3Δ met15Δ UBX2-TAP::HIS3MX6* |  |
| strain, strain background (*Saccharomyces cerevisiae*) | *yTHC* | GE Dharmacon | URA3::CMV-tTA MATa his3-1 leu2-0 met15-0 |  |
| strain, strain background (*Saccharomyces cerevisiae*) | *Sis1-DAmP* | GE Dharmacon | URA3::CMV-tTA MATa his3-1 leu2-0 met15-0 tet-SIS1 |  |
| strain, strain background (*Saccharomyces cerevisiae*) | BY4741 | GE Dharmacon | *his3Δ leu2Δ ura3Δ met15Δ* |  |
| strain, strain background (*Saccharomyces cerevisiae*) | *afg3Δ* | GE Dharmacon | *his3Δ leu2Δ ura3Δ met15Δ afg3::KanMX* |  |
| strain, strain background (*Saccharomyces cerevisiae*) | *dsk2Δ* | GE Dharmacon | *his3Δ leu2Δ ura3Δ met15Δ dsk2::KanMX* |  |
| strain, strain background (*Saccharomyces cerevisiae*) | *hlj1Δ* | GE Dharmacon | *his3Δ leu2Δ ura3Δ met15Δ hlj1::KanMX* |  |
| strain, strain background (*Saccharomyces cerevisiae*) | *hsp104Δ* | GE Dharmacon | *his3Δ leu2Δ ura3Δ met15Δ hsp104::KanMX* |  |
| strain, strain background (*Saccharomyces cerevisiae*) | *mdm10Δ* | GE Dharmacon | *his3Δ leu2Δ ura3Δ met15Δ mdm10::KanMX* |  |
| strain, strain background (*Saccharomyces cerevisiae*) | *mdm12Δ* | GE Dharmacon | *his3Δ leu2Δ ura3Δ met15Δ mdm12::KanMX* |  |
| strain, strain background (*Saccharomyces cerevisiae*) | *mdm30Δ* | GE Dharmacon | *his3Δ leu2Δ ura3Δ met15Δ mdm30::KanMX* |  |
| strain, strain background (*Saccharomyces cerevisiae*) | *mdm34Δ* | GE Dharmacon | *his3Δ leu2Δ ura3Δ met15Δ mdm34::KanMX* |  |
| strain, strain background (*Saccharomyces cerevisiae*) | *mmm1Δ* | GE Dharmacon | *his3Δ leu2Δ ura3Δ met15Δ mmm1::KanMX* |  |
| strain, strain background (*Saccharomyces cerevisiae*) | *msp1Δ* | GE Dharmacon | *his3Δ leu2Δ ura3Δ met15Δ msp10::KanMX* |  |
| strain, strain background (*Saccharomyces cerevisiae*) | *oma1Δ* | GE Dharmacon | *his3Δ leu2Δ ura3Δ met15Δ oma1::KanMX* |  |
| strain, strain background (*Saccharomyces cerevisiae*) | *pep4Δ* | GE Dharmacon | *his3Δ leu2Δ ura3Δ met15Δ pep4::KanMX* |  |
| strain, strain background (*Saccharomyces cerevisiae*) | *pim1Δ* | GE Dharmacon | *his3Δ leu2Δ ura3Δ met15Δ pim1::KanMX* |  |
| strain, strain background (*Saccharomyces cerevisiae*) | *san1Δ* | GE Dharmacon | *his3Δ leu2Δ ura3Δ met15Δ san1::KanMX* |  |
| strain, strain background (*Saccharomyces cerevisiae*) | *shp1Δ* | GE Dharmacon | *his3Δ leu2Δ ura3Δ met15Δ shp1::KanMX* |  |
| strain, strain background (*Saccharomyces cerevisiae*) | *sse1Δ* | GE Dharmacon | *his3Δ leu2Δ ura3Δ met15Δ sse1::KanMX* |  |
| strain, strain background (*Saccharomyces cerevisiae*) | *sti1Δ* | GE Dharmacon | *his3Δ leu2Δ ura3Δ met15Δ sti1::KanMX* |  |
| strain, strain background (*Saccharomyces cerevisiae*) | *ubc4Δ* | GE Dharmacon | *his3Δ leu2Δ ura3Δ met15Δ ubc4::KanMX* |  |
| strain, strain background (*Saccharomyces cerevisiae*) | *ubx3Δ* | GE Dharmacon | *his3Δ leu2Δ ura3Δ met15Δ ubx3::KanMX* |  |
| strain, strain background (*Saccharomyces cerevisiae*) | *ubx4Δ* | GE Dharmacon | *his3Δ leu2Δ ura3Δ met15Δ ubx4::KanMX* |  |
| strain, strain background (*Saccharomyces cerevisiae*) | *ubx5Δ* | GE Dharmacon | *his3Δ leu2Δ ura3Δ met15Δ ubx5::KanMX* |  |
| strain, strain background (*Saccharomyces cerevisiae*) | *ubx6Δ* | GE Dharmacon | *his3Δ leu2Δ ura3Δ met15Δ ubx6::KanMX* |  |
| strain, strain background (*Saccharomyces cerevisiae*) | *ubx7Δ* | GE Dharmacon | *his3Δ leu2Δ ura3Δ met15Δ ubx7::KanMX* |  |
| strain, strain background (*Saccharomyces cerevisiae*) | *vms1Δ* | GE Dharmacon | *his3Δ leu2Δ ura3Δ met15Δ vms1::KanMX* |  |
| strain, strain background (*Saccharomyces cerevisiae*) | *ydj1Δ* | GE Dharmacon | *his3Δ leu2Δ ura3Δ met15Δ ydj1::KanMX* |  |
| strain, strain background (*Saccharomyces cerevisiae*) | *yme1Δ* | GE Dharmacon | *his3Δ leu2Δ ura3Δ met15Δ yme1::KanMX* |  |
| strain, strain background (*Saccharomyces cerevisiae*) | *yta12Δ* | GE Dharmacon | *his3Δ leu2Δ ura3Δ met15Δ yta12::KanMX* |  |
| recombinant DNA reagent | pRS315 | (Sikorski and Hieter, 1989) | *CEN LEU2* |  |
| recombinant DNA reagent | pRS316 | (Sikorski and Hieter, 1989) | *CEN URA* |  |
| recombinant DNA reagent | pADH1-Fzo1pHA | (Cohen et al., 2008) | *CEN URA3 pADH1-FZO1HA* |  |
| recombinant DNA reagent | pMM157 | This study | *CEN LEU2 pSAM35-sam35-2HA^ts^* |  |
| recombinant DNA reagent | pMM158 | *(Metzger et al., 2017)* | *CEN LEU2 pSAM35-SAM35HA* |  |
| recombinant DNA reagent | pMM159 | *(Metzger et al., 2017)* | *CEN LEU2 pSEN2-SEN2HA* |  |
| recombinant DNA reagent | pMM160 | This study | *CEN LEU2 pSEN2-sen2-1HA^ts^* |  |
| recombinant DNA reagent | pMM190 | *(Metzger et al., 2017)* | *CEN LEU2 pFZO1-FZO1HA* |  |
| recombinant DNA reagent | pMM231 | This study | *CEN URA3 pSAM35-sam35-2HA^ts^* |  |
| recombinant DNA reagent | pMM234 | This study | *CEN URA3 pSEN2-sen2-1HA^ts^* |  |
| recombinant DNA reagent | pMM242 | This study | *CEN LEU2 pUBX2-UBX2-FLAG* |  |
| recombinant DNA reagent | pMM254 | This study | *2μ URA2 pDoa1-DOA1-FLAG* |  |
| recombinant DNA reagent | pMD1 | This study | *CEN LEU2 pSAM35-sam35-2GFP^ts^* |  |
| recombinant DNA reagent | pMD4 | This study | *CEN LEU2 pSEN2-sen2-1GFP^ts^* |  |
| recombinant DNA reagent | pMD12 | *(Metzger et al., 2017)* | *CEN URA3 pTPI-mtERFP* |  |
| recombinant DNA reagent | pSM1959 | *(Metzger et al., 2008)* | *2μ LEU2 SEC63-RFP* |  |
| recombinant DNA reagent | pSM3666 | Susan Michaelis | *2μ HIS3 pCUP1-mycUbiquitin* |  |
| antibody | anti-Sam35 (rabbit polyclonal) | Chan and Lithgow, 2008 |  | WB:1:1000 |
| antibody | anti-Sis1 (rabbit polyclonal) | Yan and Craig, 1999 |  | WB:1:1000 |
| antibody | anti-Cue1 (rabbit polyclonal) | Kostova et al., 2009 |  | WB: 1:1000 |
| antibody | anti-ubiquitin (rabbit polyclonal) | Kostova et al., 2009 |  | WB: 0.25ug/ml |
| antibody | anti-MTCO1 (mouse monoclonal) | Abcam | (Abcam Cat# ab110270, RRID:AB_10863346) | WB: 3ug/ml |
| antibody | anti-Prc1 (rabbit polyclonal) | Abcam | (Abcam Cat# ab34636, RRID:AB_725928) | WB: 1ug/ml |
| antibody | anti-GFP (mouse monoclonal) | Santa Cruz Biotechnology | (Santa Cruz Biotechnology Cat# sc-9996, RRID:AB_627695) | WB: 1:400 |
| antibody | anti-phosphoglycerate kinase 1 (mouse monoclonal) | Thermo Fisher Scientific | (Thermo Fisher Scientific Cat# 459250, RRID:AB_2532235) | WB: 0.1ug/ml |
| antibody | peroxidase-conjugated anti-HA (rat monoclonal) | Roche | 3F10  (Roche Cat# 12013819001, RRID:AB_390917) | WB: 1:1000 |
| antibody | anti-FLAG (mouse monoclonal) | Sigma-Aldrich | M2  (Sigma-Aldrich Cat# F1804, RRID:AB_262044) | WB: 1:1000 |
| antibody | anti-FLAG (rabbit polyclonal) | Sigma-Aldrich | (Sigma-Aldrich Cat# F7425, RRID:AB_439687) | WB: 1:500 |
| antibody | anti-c-myc (rabbit polyclonal) | Abcam | Abcam Cat# ab9106, RRID:AB_307014) | WB: 1:2000 |
| Sequence-based reagent | oMM84 | This paper | PCR primers | GGTCCAGTTTGCACAAGACA  CCCTGAAGAACTTCGTTCAGC  GGATCCCCGGGTTAATTAA |
| Sequence-based reagent | oMM85 | This paper | PCR primers | ACTTCATCTATATCGTT  TACATACTACAGCTTGAA  AGTGAGAATTCGAGCTC  GTTTAAAC |
| Sequence-based reagent | oMM236 | This paper | PCR primers | GGGTAAGTGTAGAAAAGTG |
| Sequence-based reagent | oMM237 | This paper | PCR primers | GCCCATGATACTTGGCTAAGG |
| ­Sequence-based reagent | oJS18 | This paper | PCR primers | ACTAAATCAACGCCGCCT |
| ­Sequence-based reagent | oJS20 | This paper | PCR primers | ATCACGCACGAAGAGGTT |
| ­Sequence-based reagent | oMM257 | This paper | PCR primers | TCATGTGTGATAGTAAG  GTGTAGAGCAGCAGAT  TTGGAGTCGGATCCC  CGGGTTAATTAA |
| ­Sequence-based reagent | oMM258 | This paper | PCR primers | ATCTAGACATTATGTGTT  TTATATGATTGCTGTAA  AAGTAGAATTCGAGCTC  GTTTAAAC |
| ­Sequence-based reagent | oMM128 | This paper | PCR primers | GAGTCTCTCGAGAAGGGAA  TGGGTCACTTGTT |
| ­Sequence-based reagent | oMM129 | This paper | PCR primers | CCAGTATCTAGACTATATTAC  CCTGTTATCCC |
| ­Sequence-based reagent | oMM130 | This paper | PCR primers | GAGTCTCTCGAGAGACT  TAGTTGAACGAGTTT |
| ­Sequence-based reagent | oJS19 | This paper | PCR primers | GAAGTGGCGCGCCTCACTGAT  GATTCGCGT |
| Sequence-based reagent | oMM240 | This paper | PCR primers | GATCCTTGATTATAAAGATG  ACGATGACAAGGATTATAAA  GATGACGATGACAAGGATTA  TAAAGATGACGATGACAAGT  AAGG |
| Sequence-based reagent | oMM241 | This paper | PCR primers | CGCGCCTTACTTGTCATCGT  CATCTTTATAATCCTTGTCAT  CGTCATCTTTATAATCCTTGT  CATCGTCATCTTTATAATCAA  G |
| Sequence-based reagent | oMM267 | This paper | PCR primers | TAGCCTCGAGGCCAAGTGAAT  AACAGCATC |
| Sequence-based reagent | oMM268 | This paper | PCR primers | TAGCGGCGCGCCTTACTTGT  CATCGTCATCTTTATAATCCT  TGTCATCGTCATCTTTATAAT  CCTTGTCATCGTCATCTTTAT  AATCGGAGAGATCGTCGAAA  ATATC |
| Sequence-based reagent | oMM19 | This paper | PCR primers | CCCATATAAATCAGCATC |
|  |  |  |  |  |
|  |  |  |  |  |

Becker, J., W. Walter, W. Yan, and E.A. Craig. 1996. Functional interaction of cytosolic hsp70 and a DnaJ-related protein, Ydj1p, in protein translocation in vivo. *Mol Cell Biol*. 16:4378-4386.

Cohen, M.M., G.P. Leboucher, N. Livnat-Levanon, M.H. Glickman, and A.M. Weissman. 2008. Ubiquitin-proteasome-dependent degradation of a mitofusin, a critical regulator of mitochondrial fusion. *Mol Biol Cell*. 19:2457-2464.

Davierwala, A.P., J. Haynes, Z. Li, R.L. Brost, M.D. Robinson, L. Yu, S. Mnaimneh, H. Ding, H. Zhu, Y. Chen, X. Cheng, G.W. Brown, C. Boone, B.J. Andrews, and T.R. Hughes. 2005. The synthetic genetic interaction spectrum of essential genes. *Nat Genet*. 37:1147-1152.

Ghaboosi, N., and R.J. Deshaies. 2007. A conditional yeast E1 mutant blocks the ubiquitin-proteasome pathway and reveals a role for ubiquitin conjugates in targeting Rad23 to the proteasome. *Mol Biol Cell*. 18:1953-1963.

Ghislain, M., A. Udvardy, and C. Mann. 1993. S. cerevisiae 26S protease mutants arrest cell division in G2/metaphase. *Nature*. 366:358-362.

Heck, J.W., S.K. Cheung, and R.Y. Hampton. 2010. Cytoplasmic protein quality control degradation mediated by parallel actions of the E3 ubiquitin ligases Ubr1 and San1. *Proc Natl Acad Sci U S A*. 107:1106-1111.

Heinemeyer, W., A. Simeon, H.H. Hirsch, H.H. Schiffer, U. Teichert, and D.H. Wolf. 1991. Lysosomal and non-lysosomal proteolysis in the eukaryotic cell: studies on yeast. *Biochem Soc Trans*. 19:724-725.

Lahiri, S., J.T. Chao, S. Tavassoli, A.K. Wong, V. Choudhary, B.P. Young, C.J. Loewen, and W.A. Prinz. 2014. A conserved endoplasmic reticulum membrane protein complex (EMC) facilitates phospholipid transfer from the ER to mitochondria. *PLoS Biol.* 12:e1001969.

Metzger, M.B., M.J. Maurer, B.M. Dancy, and S. Michaelis. 2008. Degradation of a cytosolic protein requires endoplasmic reticulum-associated degradation machinery. *J Biol Chem*. 283:32302-32316.

Metzger, M.B., and S. Michaelis. 2009. Analysis of quality control substrates in distinct cellular compartments reveals a unique role for Rpn4p in tolerating misfolded membrane proteins. *Mol Biol Cell*. 20:1006-1019.

Metzger, M.B., J.L. Scales, M.F. Dunklebarger, and A.M. Weissman. 2017. The Ubiquitin Ligase (E3) Psh1p Is Required for Proper Segregation of both Centromeric and Two-Micron Plasmids in Saccharomyces cerevisiae. *G3 (Bethesda)*. 7:3731-3743.

Nathan, D.F., and S. Lindquist. 1995. Mutational analysis of Hsp90 function: interactions with a steroid receptor and a protein kinase. *Mol Cell Biol*. 15:3917-3925.

Sikorski, R.S., and P. Hieter. 1989. A system of shuttle vectors and yeast host strains designed for efficient manipulation of DNA in Saccharomyces cerevisiae. *Genetics*. 122:19-27.

Ye, Y., H.H. Meyer, and T.A. Rapoport. 2001. The AAA ATPase Cdc48/p97 and its partners transport proteins from the ER into the cytosol. *Nature*. 414:652-656.
